# A comparison of visual outcome and rotational stability of two types of toric implantable collamer lenses (TICL) : V4 versus V4c

**DOI:** 10.1371/journal.pone.0183335

**Published:** 2017-08-28

**Authors:** Joo Hyun, Dong Hui Lim, Doo Ri Eo, Sungsoon Hwang, Eui-Sang Chung, Tae-Young Chung

**Affiliations:** 1 Department of Ophthalmology, Samsung Medical Center, Sungkyunkwan University School of Medicine, Seoul, South Korea; 2 Department of Ophthalmology, Saevit Eye Hospital, Goyang, Korea; 3 Department of Preventive Medicine, College of Medicine, The Catholic University of Korea, Seoul, South Korea; 4 Rhee’s Eye Hospital, Daejeon, South Korea; The University of Melbourne, AUSTRALIA

## Abstract

**Purpose:**

To compare the efficacy and rotational stability after implantation of two types of toric implantable collamer lenses (Toric ICL^™^(TICL);V4 and V4c, STAAR Surgical Co.)

**Study design:**

Retrospective case series.

**Methods:**

This retrospective study evaluated total 48 eyes of 48 patients who underwent the implantation with V4 and V4c TICL with a central hole; A twenty-four eyes of 24 patients with V4 TICL and 24 eyes of 24 patients with V4c TICL with a central hole. Visual acuity, manifest refraction, and intraocular pressure were evaluated before and after surgery. Rotational stability (disparity between the intended axis and achieved axis) was assessed in both groups using digital anterior segment photographs, and vector analysis was also performed.

**Results:**

Uncorrected visual acuity improved in both groups without significant difference (*P* = .111). There were no statistical differences between two groups in postoperative SE and cylindrical errors (*P* = .067 and .384, respectively). The mean value of rotation was 4.17±3.31° and 3.39±2.36° in the V4 and V4c TICL groups, respectively without significant difference (*P* = .364). Vector analysis of astigmatic correction showed no significant diffrence between two groups.

**Conclusion:**

V4 and V4c TICL have similar efficacy with regard to visual acuity and refractive outcomes and rotational stability.

## Introduction

Uncorrected astigmatism, as a common refractive error, leads to blurry, double vision. Currently, phakic intraocular lens implantation (pIOL) is widely used for correction of myopic astigmatism. The toric implantable collamer lens (TICL; STAAR Surgical, Nidau, Switzerland) has been reported as a predictable and safe option.[1–6]

V4TICL is the first model launched by the STAAR surgical company and is composed of plate-haptic collagen-copolymer materials. Since it can induce pupillary block after implantation,[7–9] V4 requires laser peripheral iridotomy preoperatively. The V4c TICL is the most recently released product and has peri-optic and central holes for viscoelastic removal and aqueous flow. This new design eliminates the need for iridotomy before surgery. But both models are still used to correct refractive errors worldwide.

Few studies have reported the visual outcomes of placement of spherical ICL with a central hole.[10,11] To our knowledge, there have been no studies that compare the visual outcomes and postoperative rotation of the two types of TICL (V4 and V4c). This study reports the clinical outcomes and rotational stability of the V4c TICL in comparison with the V4 TICL.

## Patients and methods

This retrospective study included patients who received implantation of V4 TICL or V4c TICL at Samsung Medical Center, Seoul, South Korea. V4TICL and V4cTICL implantations were performed from January 2013 to July 2014. Anterior segment photographs were used to evaluate rotational stability. For comparison, we standardized the mean follow-up period not to differ between the two groups (*P* = .869). Eyes with moderate to high myopia and astigmatism ranging from 1.00 to 5.25 D were included, with no history of ocular disease (such as cataract, glaucoma or ocular hypertension, retinopathy, uveitis, or corneal dystrophy) or surgery, no abnormal topographic findings, endothelial cell density higher than 2000/mm^2^, and anterior chamber depth greater than 2.80mm as measured by ORB scan II (Bausch & Lomb, Rochester, New York, USA). Institutional review board approval was obtained from the Samsung Medical Center Institutional Review Board, and all procedures adhered to the Declaration of Helsinki.

### Phakic intraocular lens

We used V4 and V4c models of TICL. The ICL size was decided based on the horizontal sulcus-to-sulcus diameter measured with ultrasound biomicroscopy (UBM, Model 840; Zeiss-Humphrey Instruments, San Leandro, California, USA). Sulcus-to-sulcus horizontal diameter was obtained by adding sulcus to limbus distance measured on 3- and 9 o’clock side by UBM and white-to- white corneal diameter measured by ORB scan II.[12,13] The ICL power was selected from among the recommended diopters for emmetropia as recommended by the manufacturer.

### Preoperative and postoperative evaluation

We evaluated uncorrected and best-spectacle corrected visual acuity (UCVA and BSCVA), spherical and cylindrical errors, and spherical equivalent on manifest refraction (MRSE) in all affected eyes. Based on the assumption that astigmatism of the crystalline lens was not induced after surgery, manifest refraction was performed. Slit-lamp microscopy, intraocular pressure (IOP) using non-contact tonometry, dilated fundus examination and non-contact specular microscope were performed preoperatively and at the final follow-up. White-to-white distance, keratometric value and anterior chamber depth were measured by ORB scan II preoperatively. Postoperative central vault was measured using anterior segment optical coherence tomography (Carl Zeiss Meditec AG, Jena, Germany). Full postoperative examination was performed at final visit. All preoperative and postoperative measurements are the routine evaluation protocol who underwent TICL implantation surgery. No additional evaluation was done for the sake of the study. The decision to use V4 or V4c TICL was made solely by the surgeon considering various clinical factors.

### Rotational stability

Digital anterior segment photography was used to assess rotational stability. The rotation was defined as the difference between the intended axis and the achieved axis at the final follow-up, which is similar to previous reports.[2,14] Because improper head position may induce the measurement error, we set the guide mark (conjunctival vessels) to prevent head tilting.

Diamond-shaped marks (V4 TICL) or straight lines (V4c TICL) represent the alignment axis of the TICL. Assuming that the adjusted axis was identical to the intended angle, postoperative TICL rotation was automatically measured by determining the angle between the adjusted axis and TICL alignment axis using ImageJ. One independent investigator performed the measurement.

To investigate the risk factors associated with TICL rotational stability, we analyzed preoperative MRSE, spherical and cylinder power of TICL, postoperative vault, and the amount of intraoperative adjustment angle as possible risk factors. Correlations between postoperative rotation of V4 and V4c TICL and possible risk factors were evaluated.

### Surgical technique

All procedures were performed by two experienced surgeons (E-S.C. and T-Y.C.). Two weeks before the surgery, peripheral iridotomies at the 10:30 and 1:30 areas were performed using argon and Nd:YAG laser on the eyes planning to undergo V4 TICL implantation in order to prevent pupillary block after surgery. Prior to surgery, we marked the horizontal axis (at the 3- and 9-o’clock positions) on the limbus with a 24-gauge needle under slit-lamp microscopy while the patient was in a sitting position.

In the operating room, after topical anesthesia, a paracentesis and a 3.0- to 3.2-mm main temporal incision were made. After injection of hyaluronate sodium 1% (Hyal 2000; LG Life Sciences, Seoul, South Korea) into the anterior chamber, TICL was inserted through the temporal incision using an injector. Intra-Op toric axis marker II (ASICO, Westmont, IL, USA) was applied to the eye to mark the precise axis in accordance with the recommended instruction by the manufacturer. Footplates were tucked into the posterior chamber one by one, and the axis of the TICL was adjusted with the aid of the mark. After verification of the ICL axis, viscoelastic material in the anterior chamber was removed manually using a syringe filled with balanced salt solution (BSS). To control IOP, intravenous mannitol 15% was administered postoperatively and IOP was monitored after 2-hours. Antibiotics (levofloxacin 0.5% [Cravit; Santen Pharmaceutical, Osaka, Japan] or gatifloxacin 0.3% [Gatiflo; Taejoon Pharmaceutical, Seoul, South Korea]) and fluorometholone 0.1% (Flumetholon; Santen Pharmaceutical) were used four times a day for one month following the surgery.

### Vector analysis of refractive astigmatism

The change of astigmatism was evaluated based on Alpins method of vector analysis.[15] According to this method, the following vectors were determined and calculated: targeted induced astigmatism (TIA), which represents the intended vector (including magnitude and axis) to change in cylinder; surgically induced astigmatism (SIA), which represents the actual astigmatic change induced by surgery; difference vector (DV), which represents the difference of astigmatism between thc achieved and target astigmatism; magnitude of error (ME), which means the arithmetic difference the magnitudes of the SIA and TIA; Correction index (CI), which is calculated by determining the ratio of the SIA to the TIA by dividing SIA by TIA; Index of success (IOS), which is calculated by dividing the DV by the TIA. All calculations were performed using Excel software (Microsoft Office version 2007, Mocrosoft, Corp.).

### Statistical analysis

All statistical analyses were performed with SPSS software (Version 18.0, SPSS Inc., Chicago, Illinois, USA). Differences between the V4 and V4c TICL groups were compared using Mann-Whitney test. Correlation analysis was performed with Spearman correlation analysis. Differences were considered significant when *p*-values were < .05.

## Results

A total of 48 eyes (48 patients) were included in this study, including 24 eyes of 24 patients in the V4TICL group, and 24 eyes of 24 patients in theV4cTICL group. Mean age was 31.08±7.78 (range: 18 to 45) years in V4 and 24.5±7.9 (range: 18 to 55) years in V4c TICL group, which showed statistical difference (*P* = .001). Mean follow-up was 7.64±4.87 (range: 1 to 18) months for the V4TICL group and 7.38±4.62 (range: 1 to 17) months for the V4cTICL group. Preoperative MRSE, mean refractive cylinder, UCVA and BSCVA (logMAR) of the two groups were not significantly different (*P* = .386, .740, .198, and .721, respectively). Except for one eye which underwent V4 TICL implantation, all eyes had with-the-rule astigmatism. However, preoperative IOP was significantly higher in the V4c group than the V4 group, although all values were normal (*P* = .022). Preoperative endothelial cell count was also different but all eyes had cell count over 2000/mm^2^ (*P* = .006). Baseline characteristics of the eyes in the two groups are demonstrated in Table 1.

**Table 1:** Baseline characteristics of patients who underwent implantation of a toric implantable collamer lens (TICL: V4 TICL versus V4c TICL).

	V4 TICL	V4c TICL	P-value
Number of patients (eyes)	24 (24)	24 (24)	
Mean age (range) (y)	31.08 ± 7.78 (18 to 45)	24.54 ± 7.91 (18 to 55)	0.001*
Gender (Men:Women)	9 eyes: 15 eyes	4 eyes: 20 eyes	0.193^†^
Mean follow-up (range) (month)	7.64 ± 4.87 (1 to 18)	7.38 ± 4.62 (1 to 17)	0.869*
Mean MRSE (D) (range)	-10.68 ± 2.44 (-15.68 to -5.57)	-9.91 ± 2.98 (-14.25 to -4.13)	0.386*
Mean refractive cylinder (D) (range)	2.50 ± 1.24 (1.00 to 5.25)	2.34 ± 0.76 (1.25 to 4.00)	0.740*
Mean visual acuity (range) (logMAR)			
● UCVA	1.32 ± 0.63 (0.40 to 2.00)	1.55 ± 0.48 (0.70 to 2.00)	0.198*
● BSCVA	0.06 ± 0.02 (0.00 to 0.30)	0.05 ± 0.07 (0.00 to 0.20)	0.721*
Intraocular pressure (range) (mmHg)	15.15± 3.28 (8.90 to 21.00)	17.33 ± 2.52 (13.00 to 21.50)	0.022*
Endothelial cell count (/mm^2^)	2879.46 ± 412.51	3224.33 ± 291.78	0.006*

D = diopters; MRSE = manifest refractive spherical equivalent;UCVA = Uncorrected visual acuity; BSCVA = best-spectacle corrected visual acuity

*: Mann-Whitney U test;

^†^: Chi test

### Effectiveness and safety outcomes

In the V4 and V4c TICL groups, UCVA, MRSE, and refractive cylinder improved significantly in all eyes postoperatively (*P* < .001). The mean postoperative UCVA improved to 0.04±0.06 (range: 0.00 to 0.15) and 0.01±0.07 (range: -0.10 to 0.15) logMAR, respectively, and the postoperative BSCVA was 0.01±0.02 (range: 0.00 to 0.05) and -0.03±0.08 (range: -0.20 to 0.10) logMAR, respectively. There was no statistically significant difference between the two groups (*P* = .111 and .122, respectively) (Table 2).

**Table 2:** Comparison of postoperative visual outcomes between V4 and V4c toric implantable collamer lens (TICL) groups.

Visual outcome	V4 TICL	V4c TICL	P-value*
Mean postoperative uncorrected visual acuity (logMAR) (range)	0.04 ± 0.06 (0.00 to 0.15)	0.01 ± 0.07 (-0.10 to 0.15)	0.111
Mean postoperative best-spectacle corrected visual acuity (logMAR) (range)	0.01 ± 0.02 (0.00 to 0.05)	-0.03 ± 0.08 (-0.20 to 0.10)	0.122
Efficacy index	1.06	1.09	0.251
Safety index	1.14	1.20	0.348
Mean postoperative manifest refractive spherical equivalent (D) (range)	-0.41 ± 0.48 (-1.50 to 0.56)	-0.21 ± 0.39 (-1.25 to 0.38)	0.067
Mean postoperative refractive cylinder (D) (range)	0.63 ± 0.33 (0.00 to 1.25)	0.56 ± 0.29 (0.25 to 1.25)	0.384

**Fig 1:**
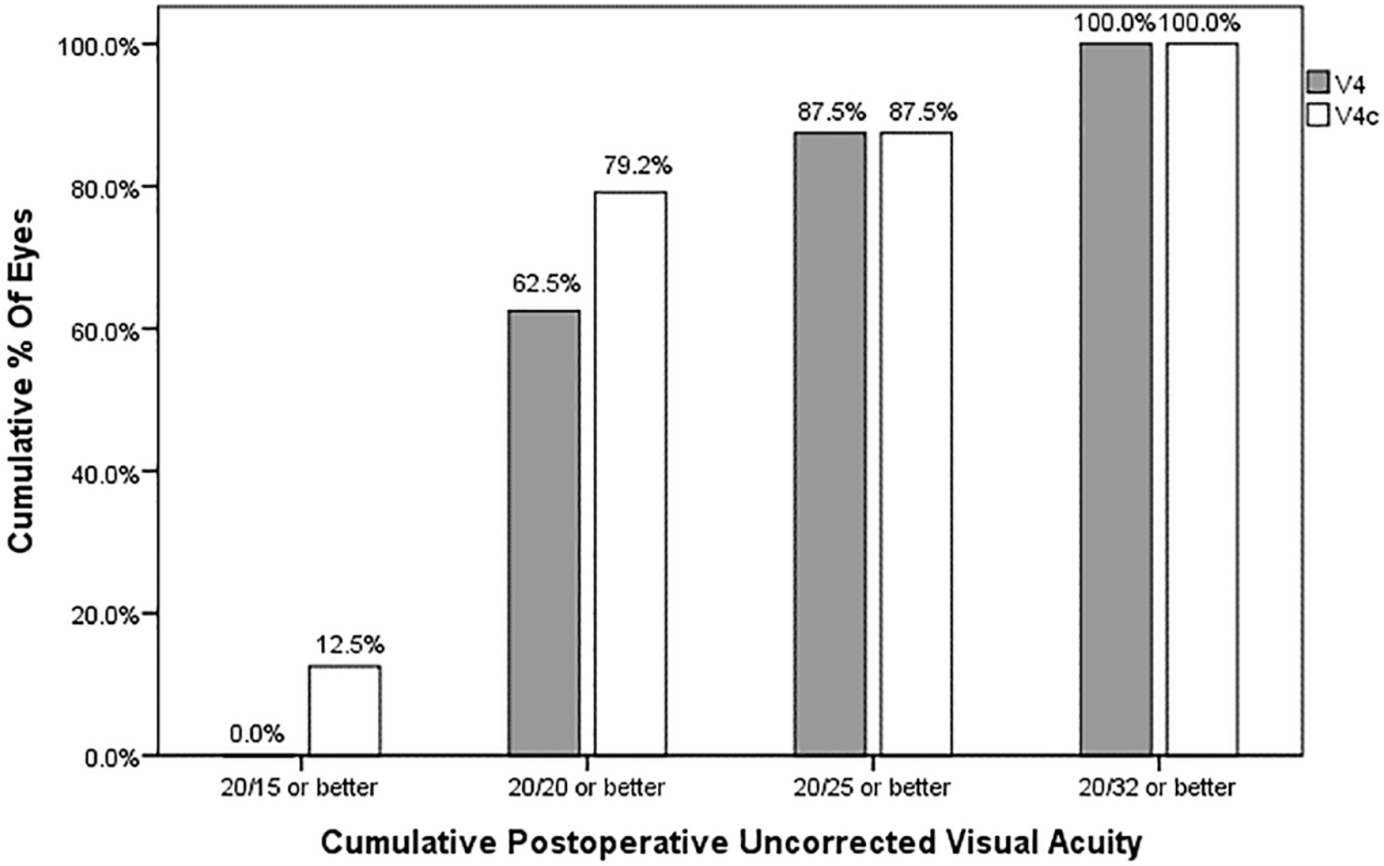
Cumulative percentages of eyes attaining specified cumulative levels of uncorrected distance visual acuity after V4 and V4c toric implantable collamer lenses (TICLs) implantations.

All eyes in both groups had postoperative UCVA of 0.15 logMAR or lower (corresponding to Snellen equivalents 20/32 or better). However, 79.2% of the V4c TICL group had UCVA of 0.00 logMAR or lower (corresponding to Snellen equivalents 20/20 or better), while 62.5% in the V4 TICL group had UCVA of 0.00 logMAR or lower (Fig 1). The difference was not statistically significant (*P* = .315, c*hi*-square test).

The efficacy indexes (mean postoperative UCVA/mean preoperative BSCVA) of V4 and V4c TICL were 1.06 and 1.09, respectively, and the safety indexes (mean postoperative BSCVA/mean preoperative BSCVA) were 1.14 and 1.20, respectively (Table 2).

### Predictability

**Fig 2:**
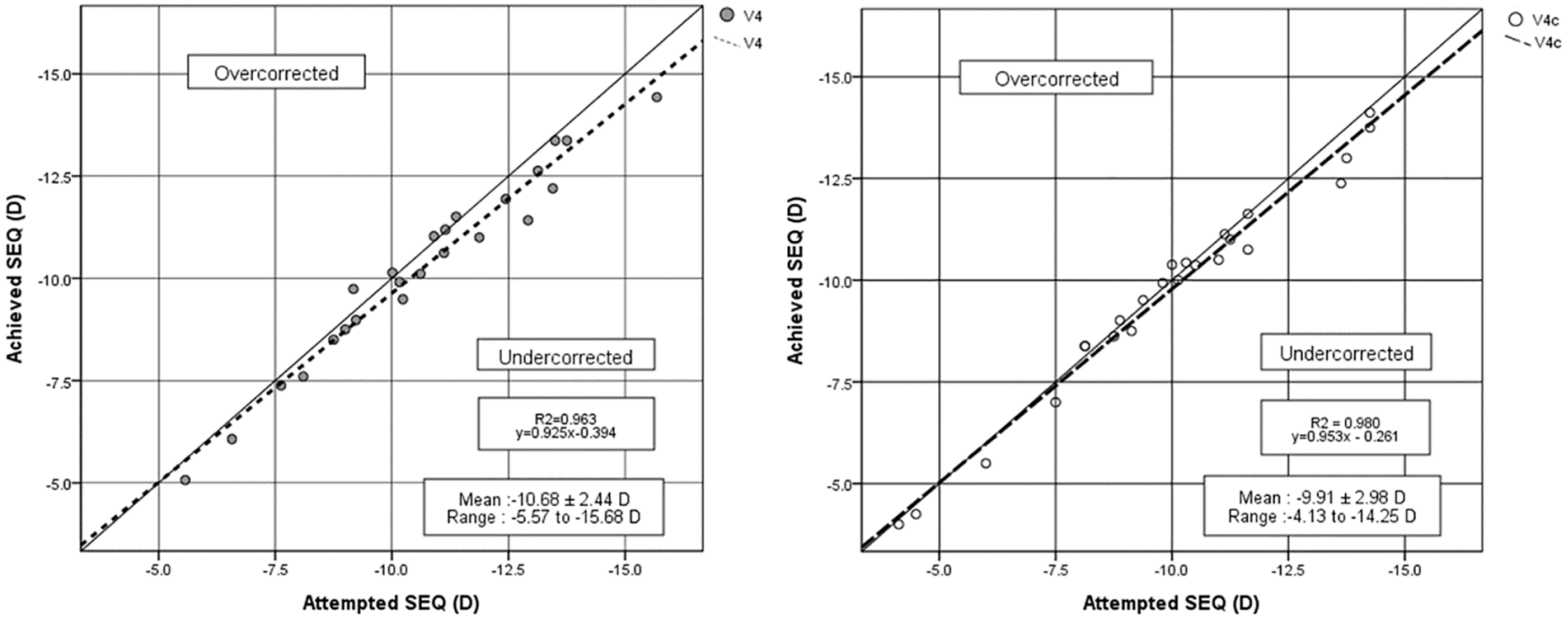
Scatter plots of the attempted versus the achieved manifest spherical equivalent (SEQ) after A) V4 and B) V4c toric implantable collamer lenses (TICLs) implantations.

The mean postoperative MRSE was -0.41±0.48 (range: -1.50 to 0.56)D in the V4 TICL group and -0.21±0.39 (range: -1.25 to 0.38) D in the V4c TICL group (Table 2). Fig 2 shows the difference in the attempted versus the achieved spherical equivalent correction. Postoperative MRSE were within ±1.5D of emmetropia in all eyes.

**Fig 3:**
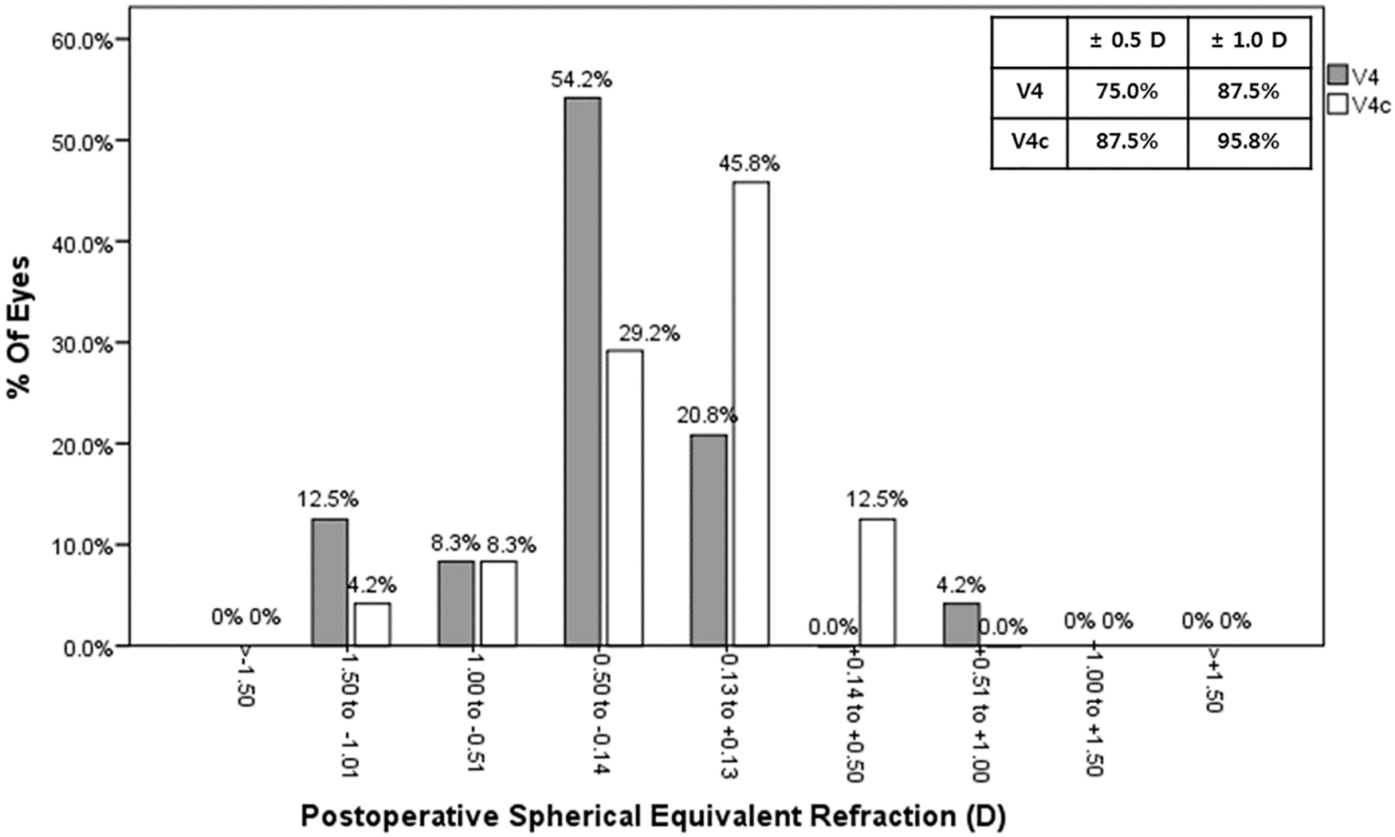
Percentages of eyes within different diopter ranges of the attempted spherical equivalent (SEQ) after V4 and V4c toric implantable collamer lenses (TICLs) implantations.

Though not significantly different, the V4c TICL group had an MRSE of ±0.5D or less in 21 of 24 eyes (87.5%) compared with 18 of 24 eyes (75.0%) in the V4 TICL group (*P* = .461, *Chi*-square test; Fig 3).

**Fig 4:**
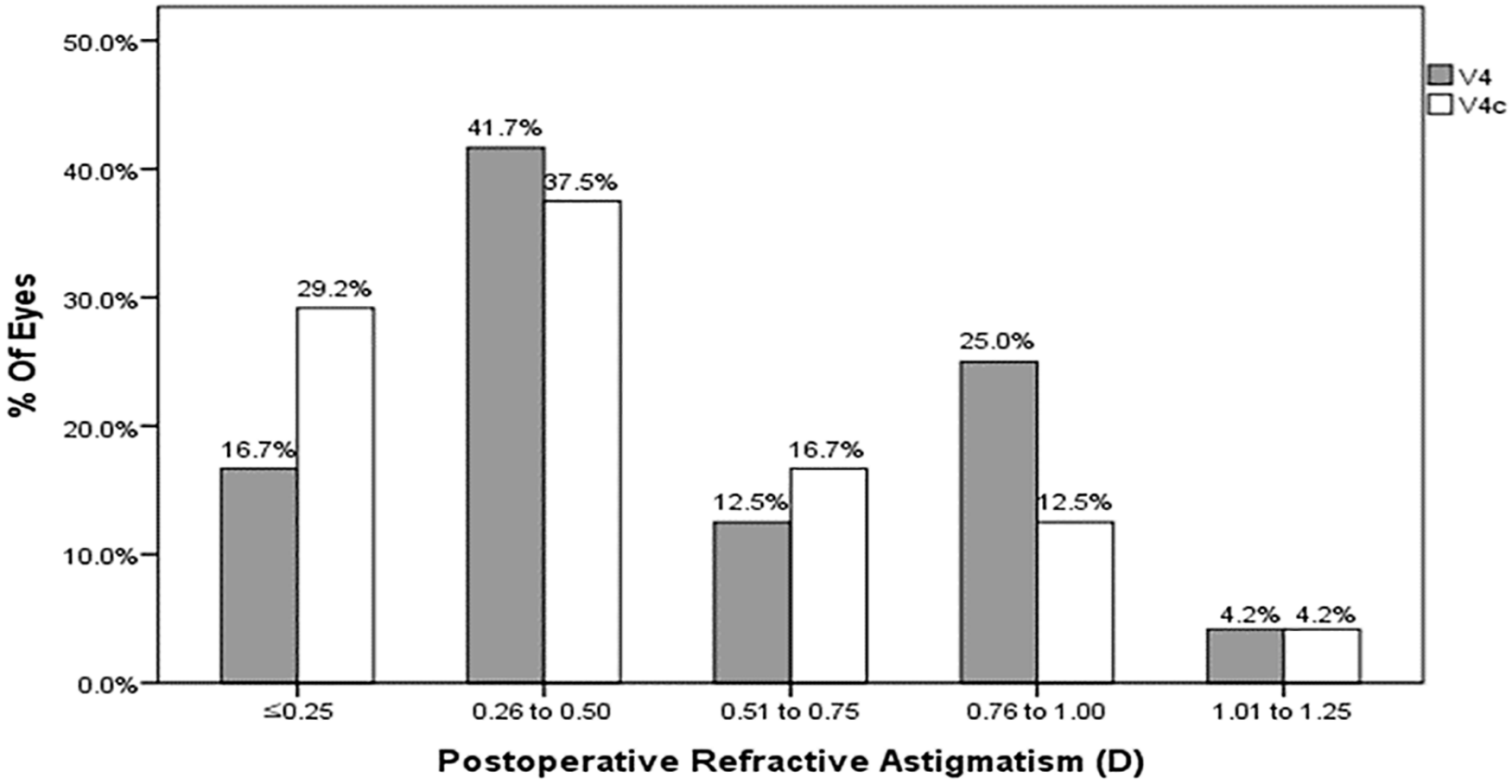
Percentages of eyes attaining specified levels of astigmatism after V4 (A) and V4c (B) toric implantable collamer lenses (TICLs) implantations.

The mean refractive cylinder was 0.63±0.33 (range: 0.00 to 1.25) D in the V4 TICL group and 0.56±0.29 (range: 0.25 to 1.25) D in the V4c TICL group, which was not significantly different (Table 2). V4 TICL and V4c TICL reduced astigmatism by 74.8% and 76.1%, respectively. All eyes achieved a refractive cylinder ≤1.25D (Fig 4).

### Rotational stability and risk factors associated with TICL rotation

**Fig 5:**
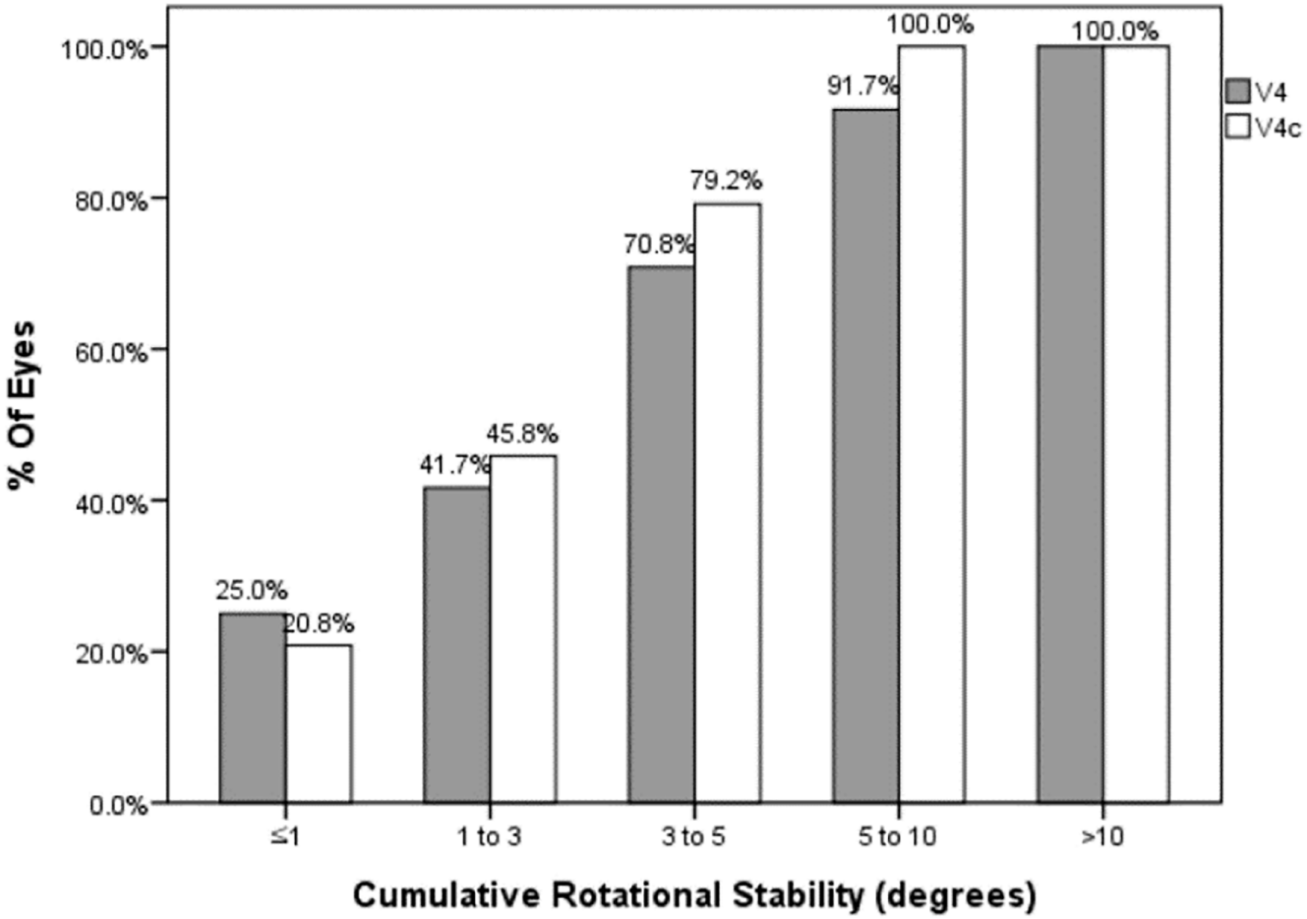
Postoperative rotational stability of the V4 and V4c toric implantable collamer lens (TICL). The angle represents the axis deviation including intraoperative misalignment of two types of TICL.

The mean value of rotation was 4.17±3.31 (0.00 to 11.00° in the V4 TICL group and 3.39±2.36 (0.31 to 9.57° in the V4c TICL group. There was no statistically significant difference (*P* = .489). Seventy-nine point two percent of eyes with V4c TICL and 70.8% of eyes with V4 TICL had rotational stability under 5 degrees until last follow up (Fig 5).

Although spherical power of the implanted TICL was more myopic in the V4 TICL group than V4c TICL group (*P* < .001), the estimated spherical power after implantation showed no significant difference (*P* = .620). Power of cylinder had no difference between the two groups (*P* = .113) as well. On the other hands, the mean absolute value of intraoperative adjustment of TICL during implantation was 7.75±6.06 (range: 0.00 to 21.00° in the V4 TICL group, which was significantly larger than 3.00±2.52 (range: 0.00 to 11.00° in the V4c TICL group (*P* = .006) (Table 3). Mean postoperative central vault was 687.50 ± 232.65μm in V4 TICL group, 674.71 ± 192.71μm in V4c TICL group, respectively. There was no significant difference between two groups (*P* = .902).

**Table 3:** Intraoperative adjustment and postoperative rotation of the toric implantable collamer lenses.

	V4 TICL	V4c TICL	*P*-value*
Power of ICL			
● Sphere (D)	-16.48 ± 3.24 (-23.00 to -9.50)	-12.27 ± 2.86 (-17.00 to -6.50)	<0.001
● Estimated Sphere (D)(Sphere*0.78)	-11.70 ± 4.53 (-17.94 to -7.41)	-12.27 ± 2.86 (-17.00 to -6.50)	0.620
● Cylinder (D)	2.83 ± 1.37 (1.00 to 6.00)	2.19 ± 0.81 (1.00 to 4.00)	0.113
Mean amount of intraoperative adjustment(degree)	7.75 ± 6.06 (0.00 to 21.00)	3.00 ± 2.52 (0.00 to 11.00)	0.006
Mean postoperative rotation (degree)	4.17 ± 3.31 (0 to 11.00)	3.39 ± 2.36 (0.31 to 9.57)	0.489
Postoperative vault (μm)	687.50 ± 232.65	674.71 ± 192.71	0.902

D = Diopter; ICL = Implantable collamer lens; TICL = Toric implantable collamer lens

*: Mann-Whitney U test

We compared the results of vector analysis based on refractive astigmatic change (Table 4). In V4 TICL group, mean magnitude of TIA was 2.31±1.24 (range: 1.00 to 5.22) D, the mean magnitude of SIA at final visit was 2.11 ± 1.09 (range: 0.75 to 4.50) D and difference vector was 0.52 ± 0.31 (range: 0.11 to 1.22) D. In V4c TICL group, mean magnitude of TIA was 2.14± 0.77(range: 0.98 to 3.91) D, the mean magnitude of SIA at final visit was 1.78± 0.70 (range: 0.51 to 3.25) D and DV was 0.40 ± 0.25 (range: 0.02 to 1.09) D. Difference between TIA and SIA was statistically significant in both groups (*P* = .03 and .00, respectively, Wilcoxon test). Tendency of slightly undercorrection was found in both groups, therefore, ME was negative, -0.21± 0.53 (range: -1.19 to 0.99) D in V4 TICL group, and -0.36 ± 0.26 (range: -0.99 to 0.13) D in V4c TICL group. CI was also under 1 in both groups, 0.95 ± 0.32 (range: 0.50 to 1.99) and 0.82 ± 0.14 (range: 0.52 to 1.07), respectively. Index of success was 0.26 ± 0.21 (range: 0.06 to 1.00) and 0.19 ± 0.11 (range: 0.01 to 0.39), respectively. All of the calculated vectors including intended and induced astimatism were not significantly different.

**Table 4:** Vector analysis and comparison of the astigmatic change of two types of toric implantable collamer lenses (TICLs).

	V4 TICL	V4c TICL	*P*-value*
TIA	2.31 ± 1.24 (1.00 to 5.22)	2.14 ± 0.77 (0.98 to 3.91)	0.837
SIA	2.11 ± 1.09 (0.75 to 4.50)	1.78 ± 0.70 (0.51 to 3.25)	0.503
DV	0.52 ± 0.31 (0.11 to 1.22)	0.40 ± 0.25 (0.02 to 1.09)	0.257
Magnitude of Error	-0.21 ± 0.53 (-1.19 to 0.99)	-0.36 ± 0.26 (-0.99 to 0.13)	0.343
Correction Index	0.95 ± 0.32 (0.50 to 1.99)	0.82 ± 0.14 (0.52 to 1.07)	0.224
Index of success	0.26 ± 0.21 (0.06 to 1.00)	0.19 ± 0.11 (0.01 to 0.39)	0.353

TIA = target induced astigmatism; SIA = surgically induced astigmatism; DV = difference vector

*: Mann-Whitney U test

There was no correlation between axis and possible risk factors, including preoperative MRSE, spherical and cylinder power of TICL, postoperative vault, and the amount of intraoperative adjustment angle in the V4 TICL (*P* = .223, *P* = .110, *P* = .443, *P* = .136, and *P* = .848, respectively) or V4c TICL(*P* = .351, *P* = .436, *P* = .091, *P* = .913, and *P* = .474, respectively) group (Table 5).

**Table 5:** Spearman’s rho correlation between postoperative rotation of toric implantable collamer lens (TICL) and possible risk factors.

	Rotational stability			
	V4		V4c	
	r	*P*-value	r	*P*-value
Preoperative MRSE	-0.258	0.223	-0.199	0.351
Power of lens				
● Sphere (D)	-0.334	0.110	-0.167	0.436
● Cylinder (D)	0.164	0.443	-0.353	0.091
Postoperative vault	0.314	0.136	0.024	0.913
Intraoperative adjustment (degree)	0.041	0.848	-0.153	0.474

MRSE = Manifest refractive spherical equivalent; D = Diopter

### Complications

Mean preoperative IOP was 15.15±3.28 (range: 8.90 to 21.00) mmHg in the V4 TICL group and 17.33±2.52 (13.00 to 21.50) mmHg in the V4c TICL group. There was a difference between the two groups in pre- and postoperative IOP (*P* = .022, .006, respectively); however, mean changes in IOP were 0.11±3.08 (range: -6.00 to 5.00) mmHg and 0.65±2.08 (-4.00 to 4.50) mmHg in the V4 and V4c TICL groups, respectively. No statistically significant difference was found (*P* = .536).

In the V4c TICL group, however, three patients revisited the hospital on the day of operation because of severe symptoms of IOP elevation, such as headache and nausea. The mean IOP was 30.67 mmHg (22, 25, 45mmHg, respectively) at that time. After manual decompression by pressing on the posterior lip of the paracentesis site, the decreased IOP was maintained until the final follow-up without anti-glaucoma medication.

Endothelial cell count was significantly different between V4c and V4 group (*P* = .006) before the surgery. However, postoperatively, mean change was not significantly different (*P* = .789). Endothelial cell count decreased by 84.46±308.94 (range: -844 to 669) in the V4 TICL group and 119.08±218.82 (range: -598 to 235) in the V4c TICL group. Other complications, such as cataract formation and pigment dispersion glaucoma, did not occur during follow-up in either group.

## Discussion

In the current study to compare visual acuity, refractive errors and rotational stability after V4 and V4c TICL implantation, the two groups showed similar visual outcomes. While V4 TICL implantation is known as a safe and effective method to correct myopic astigmatism,[2,4,14,16] visual and refractive outcomes and rotational stability of V4c TICL have not been reported. To the best of our knowledge, this is the first study to compare visual outcomes and rotational stability of V4 and V4c TICL.

The V4c TICL has both a 360-μm port in the center of the optic and two perioptic holes, whereas V4 TICL has no holes. The holes facilitate removal of viscoelastic materials and allow natural flow of aqueous humor, avoiding the need for laser peripheral iridotomy before ICL implantation. V4 ICL stored in sodium chloride (NaCl) adjusts to the internal environment and hydrates once implanted, whereas the V4c stored in BSS does not enlarge after implantation.

Alfonso *et al*.[1] have reported that V4b TICL, which is similar to V4c TICL except for the central hole, had good and highly stable visual and refractive outcome. The authors have also shown that ICL with a central hole was effective, predictable, and safe for the correction of moderate to high myopic errors.[10]

In the current study, postoperative UCVA and BSCVA were similar in these two types of TICL. All eyes in both groups had UCVA of 0.15 logMAR or lower. However, 19 (79.2%) of 24 eyes of the V4c TICL group had UCVA of 0.00 logMAR or lower, while only 15 (62.5%) of 24 eyes in the V4 TICL group had UCVA of 0.00 logMAR or lower. Although it seems to show better visual outcomes in V4c TICL group, there was no significant difference.

In terms of refractive outcome, we found that postoperative MRSE of all eyes was within ±1.5D of emmetropia, and that 21 of 24 eyes (87.5%) in V4c TICL group had MRSE of ±0.5D or less. Postoperative refractive cylinder decreased in both groups without significant difference. V4 TICL reduced astigmatism by 74.8%, and V4c TICL reduced astigmatism by 76.1%. All eyes of both groups had refractive cylinder within 1.25D. The results correspond with other studies reporting the reduction of astigmatism from 70.1% to 77.5%.[2–4]

One of the important factors that determine postoperative refractive outcome is the rotation of the implanted TICL. It has been shown theoretically and clinically that the toric intraocular lens (IOL) loses its effect on astigmatism as the lens rotates off-axis. If an IOL rotates 30 degrees off-axis, the correction effect on astigmatism will be 0, and the relationship between rotation and correction effect is fairly linear.[17,18] Theoretically, V4c TICL might have better rotational stability than V4 TICL. V4 TICL, which is stored in NaCl, adjusts to the internal environment and is enlarged about 1.05 times in the eye within 2–3 days. Because of this property, V4 TICL might be vulnerable to rotation before it fully enlarges and becomes fixed between the ciliary sulcus. V4c TICL, on the other hand, is stored in BSS and does not change in size after implantation. It should therefore be fixed between the ciliary sulcus immediately after implantation, resulting in better rotational stability.

Hence, we compared the rotational stability between V4 TICL and V4c TICL. The mean value of rotation (disparity between the intended axis and achieved axis) was greater in the V4 TICL group than the V4c TICL group, though the difference was not statistically significant (*P* = .489). Rotational stability was comparable to that in previous studies using similar measuring methods.[2,14] In addition, Hashem et *al*.[4] reported a 2.68±2.11° postoperative rotation as measured by OPD-Scan II (NIDEK co Ltd, Gamagori, Japan), and Mori et *al*.[19] reported a 4.82±6.98 (range: 0.0 to 47.2° mean rotation obtained by vector analysis. Even if we take into account the difference in measurement methods, the rotation of V4 and V4c TICL in the current study showed similar rotational stability.

In our study, the mean adjustment angle was 7.75±6.06 (range: 0.00 to 21.00° and 3.00±2.52 (range: 0.00 to 11.00° in the V4 and V4c TICL groups, respectively (*P* = .006). Mori et *al*.[19] reported that eyes with a fixation angle of 5 degrees or more were 5.6 times more likely to have rotation than eyes with an angle less than 5 degrees; however, we could not find a correlation between adjustment angle and axis deviation in either group. This statistical results probably came from small sample size or a few cases with large adjustment angle in V4 TICL group (5 cases, range: 13.0 ~ 21.0 degree).

Park et *al*.[2] reported that the spherical power of the TICL was correlated with the angle of TICL rotation. The overall height and size of the optic become thicker and smaller as TICL power becomes more negative, which could have an effect on the stability of TICL. In our study, V4 TICL had more negative power (-16.48±3.24D) than V4c TICL (-12.27±2.86D; *P* < .001). According to the manufacturer’s reports, as V4 TICL enlarges after implantation, true spherical power decreases by 0.78 times in the eye. For example, a V4 TICL of -10.0D becomes -7.8D in the eye. After considering this power change of V4 TICL, the converted spherical power of V4 TICL (Spherical power * 0.78) was not different from that of V4c TICL (*P* = .620). Therefore, there is a strong presumption that the spherical power of the two types of ICL has no effect on postoperative rotation, which is also different from previous reports. In the current study, we were unable to identify risk factors of rotational stability, including previously reported risk factors, such as the adjustment angle or the spherical power of TICL. Since ICL rotation is not fully understood and seems to involve a complicated process, further study is required to reach a definitive conclusion.

To determine the effect of the TICL on astigmatic correction, vectorial analysis was performed in our study using Alpins method. A trend toward undercorrection of astigmatism was found in both groups, some studies have also reported that trend using vectorial analysis after TICL implantation.[3, 20] Regarding efficiency of astigmatism correction, IOS which represents a relative meausre of success had no significant difference between two groups.

Mean change in intraocular pressure (IOP) was not different in the V4 and V4c TICL groups (*P* = .536). While Shimizu *et al*.[21] did not find elevation in IOP in the early postoperative period (3 to 6 hours), Gonzalez-Lopez et *al*.[22] reported an IOP increase in 5 of 100 eyes after V4c ICL implantation. They thought that the main cause of IOP elevation was obstruction of the trabecular meshwork or central hole by residual viscosurgical device. We also considered that TICL narrowed the iridocorneal angle,[23] and remaining viscoelastic material behind holes of V4c TICL might temporarily block the flow of aqueous humor. Furthermore, the affected pupil got smaller over time, and the iris covered the perioptic holes that act as another channel of aqueous flow. In this circumstance, absence of iridotomies could precipitate transient IOP elevation. Although V4c TICL eliminated the need for preoperative iridotomy, clinicians should keep in mind that IOP can increase in the early postoperative period when using this lens.

It is not clear which model is superior with regard to postoperative rotational stability. In fact, we evaluated the misalignment of ICL, not true rotation, which contains the axis deviation caused by surgeon during the surgery, as we could not collect photographs right after the surgery. Our study has a few more limitations such as a small number of cases, significantly different preoperative data between two groups (age, IOP and endothelial cell count). But we verified that the mean change in pre- and postoperative IOP and endothelial cell count was not significant.

We also did not evaluate subjective visual performances, such as glare or halo.[24] Further investigation with a larger sample size, longer follow-up, and controlled prospective nature is needed.

In conclusion, V4 and V4c TICL have similar efficacy with regard to visual acuity and refractive outcome. With respect to rotational stability, both lenses showed stable postoperative rotation. However, in case of eyes with V4c TICL, early postoperative IOP elevation should be closely observed.

## Supporting information

S1 Data Data analyzed. (XLSX)
